# Impact of United States marine corps recruit training on the stress response and menstrual function in female marine recruits

**DOI:** 10.14814/phy2.71007

**Published:** 2026-07-11

**Authors:** Lauren M. Visconti, Andrea C. Givens, Laura J. Palombo, Brenda A. Niederberger, Emily B. Kloss, Daniel W. Bennett, Lorraine P. Turcotte, Karen R. Kelly

**Affiliations:** ^1^ Warfighter Performance Department Naval Health Research Center San Diego California USA; ^2^ Leidos, Inc. San Diego California USA; ^3^ Human and Evolutionary Biology University of Southern California Los Angeles California USA

**Keywords:** menstrual cycle, military, physiological stress, training

## Abstract

This study investigated the physiological stress response and menstrual cycle (MC) function in Marine recruits during United States Marine Corps Recruit Training at the Marine Corps Recruit Depot in San Diego, CA. Female recruits (*n* = 120) completed pre‐ and post‐RT MC surveys. Saliva samples were collected at 6 timepoints (pre‐RT, weeks 1, 4, 7, 10, post‐RT) and analyzed for stress biomarkers. Urine samples were collected pre‐RT, 5 days per week during RT, and post‐RT to assess sex hormone metabolites. Linear mixed‐effects models assessed time effects (*p* < 0.05). Pre‐RT sAA levels were higher than weeks 4 (*p* < 0.001), 7 (*p* < 0.001), and 10 (*p* = 0.003). Week 1 sAA levels were greater than week 7 (*p* = 0.049) and post‐RT sAA levels were greater than week 4 (*p* = 0.011), 7 (*p* < 0.001), and 10 (*p* = 0.046). Cortisol at weeks 1 (*p* = 0.004) and 4 (*p* < 0.001) was greater than week 10. Before RT, 85% reported regular MCs; during RT, 53% reported irregularity. Urine measures indicated 92% experienced MC disruption, 87.5% within the first cycle. Recruits experienced heightened stress early in RT but adapted as training progressed. Most recruits exhibited MC irregularities, with disruption occurring in the first cycle, indicating heightened hypothalamic–pituitary‐ovarian axis sensitivity to military training stress.

## Graphical abstract

Graphical overview of methods and major outcomes describing the stress response and menstrual function in female recruits during United States Marine Corps Recruit Training.

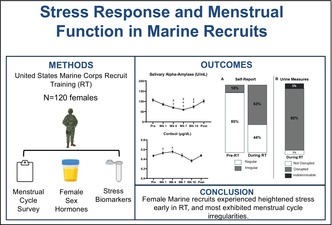

## 1 INTRODUCTION

In recent years, the number of women engaging in historically male military roles has grown (Le Menestrel et al., 2019). In 2021, the first company of female Marine recruits completed USMC Recruit Training (RT) at Marine Corps Recruit Depot in San Diego CA (MCRD‐SD), which is a rigorous 13‐week military training program that prepares aspiring Marines for the physical and operational demands endured in combat. To date, there is limited information on the impacts of entry‐level military training on female warfighter physiology, given their recent integration into combat roles (Nindl et al., 2016; Schram et al., 2022).

During USMC RT, Marine recruits are exposed to an array of stressors, including increased physical training, heightened cognitive demand, and community sleeping. The Naval Health Research Center (NHRC) recently reported heightened workload (10–12 miles/day) and determined musculoskeletal injury (MSKI) rates in female Marine recruits (Jensen et al., 2019; Schram et al., 2022) undergoing this type of training. While physical training is essential for improving muscular strength and cardiovascular endurance for operational performance, the compounding effects of chronic stress exposure coupled with insufficient recovery experienced during intensive military training can result in high allostatic load (Tait et al., 2022; Taylor et al., 2007; Visconti, Palombo, et al., 2024; Visconti, Turcotte, et al., 2024). This physiological burden accrues when stress load exceeds the body's ability to optimally cope (Sonino et al., 2023).

High allostatic load is associated with disruption of several neuroendocrine systems including the sympatho‐adrenal medullary (SAM) system (De Pero et al., 2021), hypothalamic–pituitary adrenal axis (HPAA) (Ojanen et al., 2023; Tait et al., 2022; Taylor et al., 2007; Visconti, Palombo, et al., 2024; Visconti, Turcotte, et al., 2024), and hypothalamic pituitary gonadal axis (HPGA) (Ojanen et al., 2023; Tait et al., 2022; Taylor et al., 2007; Visconti, Turcotte, et al., 2024) in military populations undergoing training. The physiological stress response is governed by both the SAM system and the HPAA. The SAM system constitutes a network of nerves that stimulates a cascade of events leading to the production of norepinephrine (NE) and epinephrine (EPI) (Cryer, 1980). Among many other functions, NE binds with receptors on the salivary gland cells in the mouth stimulating the release of salivary alpha‐amylase (sAA), which serves as a biomarker for the SAM system activation (Nater & Rohleder, 2009). The HPAA is a complex system consisting of the hypothalamus, pituitary gland, and adrenal glands that results in the production of cortisol, the body's primary stress hormone (Oyola & Handa, 2017), from the zona fasciculata cells of the adrenal cortex (Sheng et al., 2021). The SAM system acts quickly and is responsible for eliciting the “fight or flight” response (Nater & Rohleder, 2009), while the HPAA is slower to respond (Oyola & Handa, 2017). Nonetheless, both systems work synergistically to combat stress and return the body to a homeostatic state.

Moreover, the HPGA is a neuroendocrine system that regulates reproductive function, sex hormone production, and fertility, and is referred to as the HP ovarian axis (HPOA) in females (Oyola & Handa, 2017). More specifically, the HPOA regulates the menstrual cycle (MC), which constitutes the cyclical fluctuations in female sex hormones that occur to prepare the body for potential pregnancy (Hall, 2019). The HPAA and HPOA work synergistically to maintain physiological homeostasis and well‐being. Chronic overactivation of the body's stress systems (i.e., SAM system and HPAA) is associated with suppression of the HPOA, which is reflected by elevated stress biomarkers coupled with MC dysfunction (Corradi et al., 2016; Heck & Handa, 2019; Oyola & Handa, 2017). The relationship between training stress, energy imbalance, and MC dysfunction in women has been well‐studied within the context of Relative Energy Deficiency in Sport (RED‐S), which is a multifactorial syndrome characterized by symptoms such as endocrine disruption, heightened injury risk, and compromised performance in athletic populations (Areta et al., 2020; Logue et al., 2020; Wasserfurth et al., 2020). HPOA disruption is one of the most prominent hallmarks of RED‐S in women, which may result in irregular periods, anovulation, and / or functional hypothalamic amenorrhea (FHA; absence of menstrual periods for 3+ months in previously menstruating women) (Areta et al., 2020; Logue et al., 2020; Wasserfurth et al., 2020).

When applied to military populations, RED‐S is referred to as Relative Energy Deficiency in Military (RED‐M) which extends beyond athletics to operational training environments (Constantini et al., 2024). RED‐M has been predominately studied in male military populations (Garron & Klein, 2023), while data on female operators is limited (Givens et al., 2023). Nonetheless, prior investigations have documented menstrual disturbances in 53% (Gifford et al., 2017) and 86% (Bozzini et al., 2023) of women undergoing rigorous military training. Additionally, Popp et al. (2024) observed disruptions in MC hormones in women engaging in US Army Basic Combat Training, even in those who reported having normal MCs (Popp et al., 2024). These data underscore the inconsistencies between self‐reported menses and physiological biomarkers of reproductive function. Therefore, it is imperative to assess MC hormones in conjunction with self‐reported bleeding to gain greater insight on the impact of military training on the reproductive health of female operators. Moreover, given the interplay between the body's stress systems (i.e., SAM system and HPAA) and the HPOA, quantifying stress biomarkers together with MC hormones is advantageous for assessing the effects of operational training on female warfighter physiology. Therefore, the purpose of this study was to quantify stress biomarkers, female sex hormones and MC status in female Marine recruits during RT at MCRD‐SD. Information on the physiological impacts of USMC RT on female military personnel is paramount for optimizing performance, operational readiness, and overall health in novice operators.

## 2 MATERIALS AND METHODS

### 2.1 Participant recruitment

A total of 120 female Marine recruits from three separate cohorts at MCRD‐SD volunteered to participate in this study. Prior to the initiation of the study, Marine recruits were briefed by research staff. Voluntary written and verbal consent was obtained from all participants. The study protocol is in accord with the Declaration of Helsinki and was approved by the Naval Health Research Center Institutional Review Board in compliance with all applicable federal regulations governing the protection of subjects NHRC.2020.0008.

### 2.2 Baseline assessment

Participants' anthropometric measurements (i.e., height [cm.], weight [kg.] and body fat percentage [BF%]) were assessed in the morning between 0700 and 0800, having been overnight fasted 2 days prior to RT. Height and weight were measured with a stadiometer to the nearest 0.1 cm (SECA North America, Chino, CA, USA). Body weight and BF% were assessed with an InBody 770 (InBody USA, Cerritos, CA, USA).

### 2.3 USMC recruit training

USMC RT is a 13‐week entry‐level training program where both male and female Marine recruits undergo intensive training that consists of 3 weeks of administrative processing and 10 weeks of tactical training. USMC RT is divided into four phases (P1–4), each lasting approximately 3–4 weeks. During the first phase (P1), recruits undergo preemptive health and fitness screening and are equipped with foundational knowledge and skill sets that they will build upon throughout the course of training. Training load during this phase is low relative to subsequent phases. The initiation of tactical training begins during the second phase (P2) of USMC RT, whereby recruits experience an increase in training volume and undergo combat water survival training, physical and combat conditioning, martial arts training, and academic instruction. The third phase (P3) of RT is the final and most demanding phase of tactical training, whereby Marine recruits learn fundamentals of marksmanship, undergo basic warrior training, hone field skills, and complete the Crucible. The Crucible is a 54‐h day and night test where Marine recruits are assessed on their ability to perform under stressful conditions with limited food and sleep. Upon completion of the Crucible, recruits enter phase 4 (P4), which includes out‐processing tasks such as final physical and academic examinations, leadership discussion, and graduation.

### 2.4 Saliva sampling

Research staff collected saliva samples from participants after an overnight fast (between 0500 and 0600) at the following time points: (Le Menestrel et al., 2019) pre‐RT (2 days prior to the start of RT), (Schram et al., 2022) week (week) 1 (P1), (Nindl et al., 2016) week 4 (P2), (Jensen et al., 2019) week 7 (P3), (Visconti, Palombo, et al., 2024) week 10 (end of P3), and 48 h post‐RT. Participants were instructed by research staff to place a cotton swab in the back corner of their mouth for at least 3 min until fully saturated with saliva. Saturated cotton swabs were placed in corresponding de‐identifiable tubes labeled with participant ID numbers, and immediately placed in a temperature‐sensitive and validated specimen transport cooler (−20°C) and transported to Salimetrics by research staff. Samples were stored in a −80°C freezer until further analysis (within 1 week of collection). Saliva samples used to assess steroid hormones and sAA remain viable for several years when stored in a −80°C freezer (Pachimsawat & Jantaratnotai, 2026).

### 2.5 Urine sampling

Urine samples were collected upon waking (between the hours of 0500 and 0600) 1 day prior to RT, 5 days per week during RT (Monday–Friday), and 1 day post‐RT, totaling 58 samples for each participant. Participants were instructed to collect the first morning void in a de‐identifiable sanitary collection container (provided by research staff) labeled with participant ID numbers. Upon collection, sealed collection containers were placed in a standard refrigerator (4.4°C) until further processing by research staff. Collection cards containing sterile filter paper (labeled with participant ID, date, and time of sampling) were placed in corresponding urine containers and removed once fully saturated with urine. Saturated collection cards were draped over sterile drying racks for 6 h and then placed into sealed plastic bags by research staff. Urine collection cards were refrigerated at 4.4°C until further analysis.

### 2.6 Saliva analysis

Saliva samples were analyzed by Salimetrics LLC (State College, PA, USA), a global leader in salivary bioscience. Saliva was analyzed for sAA levels using the Salimetrics Salivary Alpha‐Amylase Kit (Cat. No. 1–1902) via kinetic enzyme assay, and cortisol concentrations using the Salivary Cortisol Assay Kit (Cat. No. 1–3002) via enzyme‐linked immunosorbent assay (ELISA). All samples were analyzed in duplicate (2 technical replicates per sample) to assess technical variability and improve measurement reliability. Mean values of each duplicate were used for downstream analysis.

### 2.7 Urine analysis

Urine samples were analyzed for female sex hormone metabolite (pregnanediol glucuronide [PdG], estrone‐3‐glucuronide [E3G], and luteinizing hormone [LH]) concentrations via gas chromatography, mass spectrometry (GC–MS/MS) by ZRT Laboratory (Beaverton, OR, USA), a CLIA certified diagnostic laboratory in hormone and wellness testing. GC–MS/MS is a validated method for measuring female sex hormone metabolite concentrations in urine (Alladio et al., 2021; Moon et al., 2011; Newman et al., 2019). All samples were analyzed in duplicate (2 technical replicates per sample) to assess technical variability and improve measurement reliability. Mean values of each duplicate were used for downstream analysis.

### 2.8 Menstrual cycle survey

All female recruits voluntarily completed a Reproductive Health History Questionnaire (Givens et al., 2023) at the start of RT to gather information regarding age at menarche, average MC length and number of days between periods, regularity over the past 12 months, all past and current use and methods of birth control, and the first day of their last period. At the end of RT, participants voluntarily completed a follow‐up questionnaire to gather information on MC activity during RT, specifically the number of periods experienced and bleeding length (days) per period.

### 2.9 Statistical analysis

#### 2.9.1 Salivary biomarkers

All statistical analyses were performed using R.4.5.0 (R Core Team, 2025). Outliers were evaluated using a combination of statistical and robust diagnostic approaches, including Grubbs' and Dixon's tests for extreme values, median absolute deviation (MAD) thresholds, boxplot‐based IQR criteria, and *z*‐score screening. A linear mixed‐effects model (LMM) was used to evaluate sAA and cortisol concentrations across 6 time points (pre‐RT, week 1, 4, 7, 10, and post‐RT). Residual normality was assessed via quantile‐quantile (Q‐Q) plots. Fixed effects included time, while random intercepts were included for participants to account for within‐subject comparisons due to repeated measures. This approach accounts for missing data under the assumption of missing at random (MAR) and leverages all available data without requiring complete cases. The model was fit using the lme4, lmerTest, and emmeans packages in R with restricted maximum likelihood estimation (REML). Significance of fixed effects was assessed via Type III ANOVA using Satterthwaite's method for degrees of freedom. To control Type I error due to multiple pairwise comparisons between time points (pre‐RT, week 1, 4, 7, and 10, and post‐RT), a Bonferroni correction was applied. The significance threshold was adjusted by dividing the conventional alpha level (*α* = 0.05) by the number of comparisons performed. This conservative approach reduces the likelihood of false positives when conducting multiple hypothesis tests. A *p*‐value of <0.05 was regarded as statistically significant. All data are represented as estimated marginal means (EMM) ± standard error of the mean (SEM).

#### 2.9.2 Menstrual cycle survey data

Descriptive statistics (i.e., mean and SEM) were calculated for each MC variable prior to and during RT. MC variables prior to RT include: (Le Menestrel et al., 2019) cycle length (days), period length (days), and number of periods within the past 3, 6, 9, and 12 months. Whereas MC variables during RT include number of periods that occurred throughout 57 days of training and period length (days).

To evaluate whether USMC RT resulted in changes in average period length, paired‐samples *t*‐tests were conducted, as this design accounts for within‐subject comparisons. Assumptions of normality were evaluated via inspection of residual distributions. Wilcoxon signed‐rank tests were used when data violated normality assumptions. A *p*‐value of <0.05 was regarded as statistically significant.

### 2.10 Menstrual cycle status

Pre‐RT self‐reported MC data was further categorized as being “regular” or “irregular” (>35 or <24 days between periods (Hampson, 2020) and / or having missed ≥3 consecutive periods in 12 months (Lord et al., 2024)). During RT (57‐day period) MC function was classified as being “regular” or “irregular” based on both post‐RT survey data (i.e., self‐reported bleeding) and urinary metabolite concentrations (E1G/Cr [ng/mg], PdG/Cr [ng/mg], and LH/Cr [mIU/mg]). Post‐RT survey data including number of periods experienced and bleeding length (days) per period during RT along with trends in urinary metabolite concentrations for each participant were inputted into individual Microsoft Excel (v2602, Microsoft Corp., WA, USA) spreadsheets. Rolling 5‐day averages for urinary metabolites for each participant were calculated in excel and graphically represented. Since participant's entered RT on various days of their MC, MCs were anchored based on the participant's first day of their last period, as reported in the pre‐RT Reproductive Health Survey. Graphed data (self‐reported bleedings days and urinary metabolite concentrations) were assessed by two separate research personnel (independently) to determine MC status (i.e., “regular” or “irregular”) during RT. To be categorized as “regular”, profiles had to meet the following criteria: (Le Menestrel et al., 2019) presence of an LH spike and concomitant rise in E1G, (Schram et al., 2022) subsequent rise in PdG within 2 days of LH spike, (Nindl et al., 2016) self‐reported period ≥3 days in length, and (Jensen et al., 2019) bleeding occurring at least 11 days following a spike in LH (Allaway et al., 2016). Participants who did not meet all criteria were categorized as “irregular” (Allaway et al., 2016). In the event of rater disagreement, participant data were evaluated by a third rater. If a categorization of “regular” or “irregular” could not be determined with the addition of a third rater, participant data were deemed as “indeterminable.” Participants missing ≥4 consecutive days of urine data for ≥1 metabolite (E1G, PdG, or LH), due to no sample or concentrations below the detectable limit, were excluded from the analysis (Allaway et al., 2016). For participants who were categorized as “irregular” for ≥1 cycle during RT, onset of MC irregularity (i.e., during their first or second MC) was assessed. Determination of a participant's first cycle during RT was based on the first day of their last menstrual period along with their average bleeding duration and cycle length (from the pre‐RT Reproductive Health Survey). The end of participant's first cycle and beginning of their second cycle was based on their first day of bleeding reported during RT.

## 3 RESULTS

### 3.1 Participant characteristics

A total of 105 female Marine recruits completed the full 13 weeks of RT (age: 19 ± 2 years, height: 161.8 ± 7.1 cm, weight: 61.1 ± 9.1 kg, body fat: 25.8 ± 6.5%). Fifteen Marine recruits did not complete the full 13 weeks of RT and were excluded from further analysis.

### 3.2 Stress biomarkers

**FIGURE 1:**
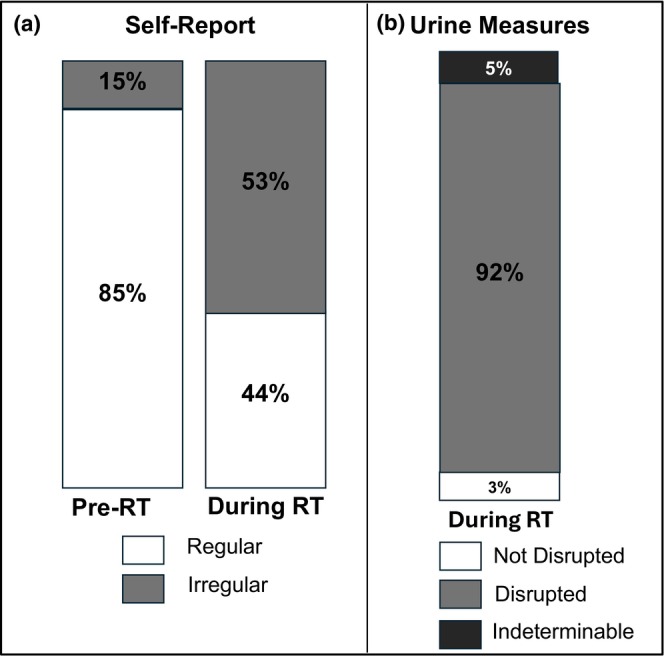
Menstrual Cycle Status in Female Marine Recruits. (a) Percentage of Marine recruits reporting regular (*n* = 52) and irregular (*n* = 9) menstrual cycles Pre‐RT (total *n* = 61). Percentage of Marine recruits reporting regular (*n* = 29) and irregular (*n* = 32) menstrual cycles during RT (total *n* = 61). (b) Percentage of not disrupted (*n* = 2), disrupted (*n* = 56), and indeterminable (*n* = 3) menstrual cycle status in Marine recruits during RT (*n* = 61). All values are represented as percentages.

Grubbs' and Dixon's tests revealed no statistically significant outliers in stress biomarkers; therefore, all data were retained for analyses. A linear mixed model revealed a significant time effect for sAA (*F*[5, 428.51] = 9.48, *p* < 0.001; Figure 1a) and cortisol (*F*[5, 490.7] = 4.32, *p* < 0.001; Figure 1b) concentrations throughout RT. Pre‐RT sAA levels (107.2 ± 6.0 U/mL) were 48%, 75%, and 48% higher than week 4 (72.5 ± 6.4 U/mL; *p* < 0.001), 7 (61.1 ± 6.6 U/mL; *p* < 0.001), and 10 (72.2 ± 8.2 U/mL; *p* = 0.003), respectively. sAA levels at week 1 (86.5 ± 6.5 U/mL) were 42% greater than at week 7 (*p* = 0.049). Moreover, post‐RT sAA levels were 40%, 66%, and 41% greater than concentrations at week 4 (*p* = 0.011), 7 (*p* < 0.001), and 10 (*p* = 0.046). Furthermore, cortisol levels at week 1 (0.53 ± 0.03 μg/dL; *p* = 0.004) and 4 (0.56 ± 0.03 μg/dL; *p* < 0.001) were 43% and 51% greater compared to week 10 (0.365 ± 0.03 μg/dL).

### 3.3 Menstrual cycle survey data

**FIGURE 2:**
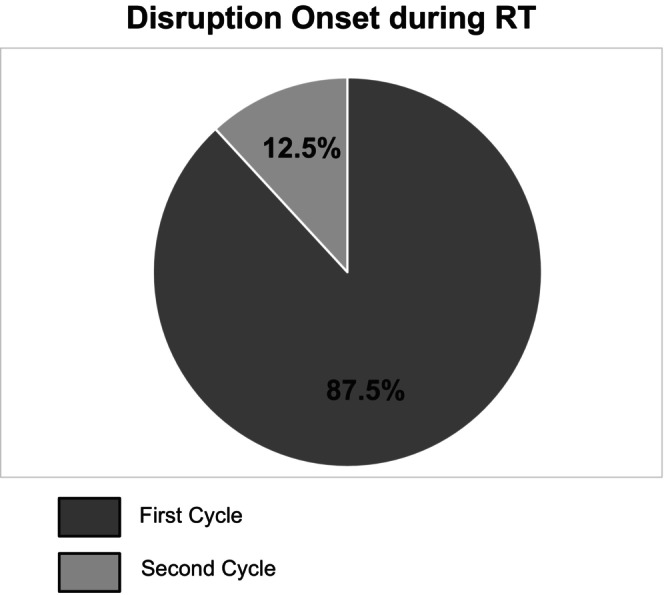
Onset of Menstrual Cycle Disruption during RT in Female Marine Recruits Categorized as having MC Disruption. Percentage of Marine recruits with first cycle disruption (*n* = 49) and second cycle disruption (*n* = 7; total *n* = 59). All values are represented as percentages.

Of the 120 female Marine recruits who volunteered to participate, 61 met inclusion criteria for MC analyses. Participants excluded from analyses did not complete the full 13 weeks of RT (*n* = 15) or had incomplete urinary hormone data (*n* = 44; i.e., missing ≥4 consecutive days of data for at least one urinary metabolite due to no sample or concentrations below the limit of detection). Female Marine recruits reported regular MCs (~1 per month) and period lengths (~5 days [within 3–7 days]) (Hall, 2019) 3, 6, 9, and 12 months prior to RT (Table 1). During RT, female Marine recruits reported having ~2 MCs within the 57‐day training period and an average period length of ~4 days. A paired‐samples *t*‐test revealed a significant difference between period length prior to RT (5 ± 1 day) relative to during RT (4 ± 2 days; *p* < 0.001; Table 1). Prior to RT, 85% (*n* = 52) of recruits reported having a regular MC, while 15% reported having an irregular MC (*n* = 9). During RT, 44% (*n* = 29) of recruits reported having regular MCs, while 53% (*n* = 32) documented having irregular cycles (total *n* = 61; Figure 2a).

**TABLE 1:** Menstrual Cycle Status (*n* = 61).

	Mean ± SD	*p*‐Value
Prior to RT		
Cycle Length (days)	29 ± 9	
Periods (within the past 3 months)	3 ± 1	
Periods (within the past 6 months)	6 ± 1	
Periods (within the past 9 months)	9 ± 2	
Periods (within the past 12 months)	12 ± 2	
Period Length (days)	5 ± 1	
During RT		
Periods (within 57 days)	2 ± 2	
Period Length (days)	4 ± 2^a^	*p* < 0.001

Abbreviations: RT, recruit training; SD, standard deviation.

^a^ Significantly different from prior to RT (*p* < 0.001).

### 3.4 Urinary female sex hormone metabolites

**FIGURE 3:**
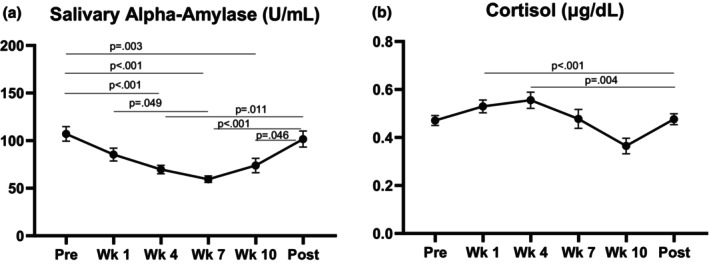
(a) Salivary Alpha‐Amylase (sAA; U/mL) and (b) Cortisol (μg/dL) concentrations at pre, weeks 1, 4, 7, and 10, and post. sAA (U/mL) and Cortisol (μg/dL) were analyzed using a linear mixed‐effects model with time as a fixed effect. A significant time effect was observed for sAA (*F*[5, 428.51] = 9.48, *p* < 0.001; [A]) and cortisol (*F*[5, 490.7] = 4.32, *p* < 0.001; [B]). All data are represented as estimated marginal means (EMM) ± standard error of the mean (SEM).

Urinary female sex hormone metabolites were analyzed for a total of 61 recruits. Urine measures indicated that 3% (n = 2) of recruits did not have disrupted MCs during RT (“not disrupted”), 92% (*n* = 56) exhibited MC disruption (i.e., deviation from an individual's regular menstrual pattern), and 3% (*n* = 3) were indeterminable (Figure 2b). For recruits who experienced MC disruption (*n* = 56), onset of disruption occurred during the first cycle in 87.5% (*n* = 49) of recruits, whereas 12.5% (*n* = 7) experienced disruption during their second cycle (Figure 3).

## 4 DISCUSSION

This study investigated the effects of entry‐level USMC‐RT on the physiological stress response, female sex hormone metabolites and MC status in female Marine recruits. Major findings demonstrate low levels of sAA during week 4, 7, and 10, relative to pre‐ and post‐RT, which is reflective of heightened anticipatory stress as Marine recruits prepare for RT (pre) and graduation (post), and SAM system recovery during training. Conversely, cortisol levels were greater at week 1 and 4 relative to week 10, which suggests elevated HPAA activity during the initial phases of training. Taken together, these data reflect the interplay between the SAM system and HPAA. Moreover, both self‐reported and urine measures demonstrated MC disruption during RT in female Marine recruits.

High levels of sAA prior to RT (pre‐RT) were observed relative to week 4, 7, and 10, which may be suggestive of anticipatory stress as recruits prepare for training. Additionally, sAA concentrations documented at week 1 were greater than week 7. sAA follows a diurnal rhythm, with levels lowest at waking and increasing throughout the day (Out et al., 2013). Morning concentrations reflect overnight recovery and basal SAM system activity, which regulates cardiovascular and metabolic functions (Out et al., 2013). Elevated morning sAA has been linked to poor sleep quality and circadian disruption of the SAM system, indicating heightened sympathetic tone upon waking (Out et al., 2013). Female recruits' low concentrations of sAA, seen at time points during RT (week 4, 7, and 10), mirror similar trends observed in Marines during Mountain Warfare Training Exercise (MTX), whereby sAA levels were higher before MTX relative to mid‐ and post‐MTX (Stein et al., 2024). Taken together, these findings may be suggestive of physiological adaptations in SAM system regulation that may arise from frequent stress exposure in military populations undergoing training. Furthermore, post‐RT sAA levels were greater than those at week 7 and 10, and similar to concentrations demonstrated prior to RT (pre‐RT). This may, in part, be due to stressors including final inspections, administrative tasks, and final evaluations as Marine recruits prepare for graduation.

Cortisol levels were elevated at week 1 and 4 compared to week 10, which were expected as recruits are exposed to an array of unfamiliar stressors characteristic of military settings and an increase in training volume. In contrast to the diurnal rhythm of sAA, cortisol levels are high in the morning and gradually decrease throughout the day (Heaney et al., 2012). Moreover, cortisol is reflective of chronic stress exposure and, thus, morning concentrations have been shown to increase during military training (Ojanen et al., 2018; Szivak et al., 2018; Taylor et al., 2007). However, there were no differences in cortisol levels at week 7 compared to all other time points (pre‐RT, week 1, 4, 7, 10, and post‐RT) and low levels at week 10 relative to week 1 and 4. In a prior NHRC study conducted in Marines during MTX, an increase in cortisol levels from pre‐ to mid‐MTX (approximately the halfway point of a 10–14 training period) was reported (Out et al., 2013). Given USMC RT is longer in duration than MTX (13 weeks total [including in and out‐processing]), the lack of changes in cortisol exhibited at week 7 and low levels documented at week 10 may suggest HPAA adaptation to chronic stress exposure in Marine recruits as training proceeds. However, in addition to training duration, it is important to note that training activities and environmental conditions differed between these two studies, which could render differential impacts on warfighter physiology.

Survey data on MC status prior to RT obtained self‐reported cycle length, menses length, and number of menses reported within the past 3‐, 6‐, 9‐, and 12‐months preceding training and indicates that participants were experiencing regular MCs. In particular, average cycle length reported was 29 ± 0.84 days, which is within the generally healthy cycle length of 21–35 days (Hall, 2019). Furthermore, number of menses reported within the past 3 (3 ± 0.09 cycles), 6 (6 ± 0.14 cycles), 9 (9 ± 0.17 cycles), and 12 (12 ± 0.32 cycles) months, along with an average menses length of 5 ± 0.14 days (3–7 days is considered healthy (Hall, 2019)), signify that participants were experiencing normal menstrual function leading up to RT. Marine recruits who reported menstruating during RT had 2 ± 0.12 periods within 57 days (~ 2 months) of training, which is normal. Menses length in menstruating participants decreased from 5 ± 0.14 to 4 ± 0.18 days during RT. While this reduction in self‐reported period length from 5 to 4 days during RT did reach statistical significance, 4 days of bleeding still within 3–7 days and is, therefore, still within normal ranges (Allaway et al., 2016). Additionally, 85% and 15% of Marine recruits reported regular and irregular MC function prior to RT, respectively. During RT, 44% and 53% of recruits reported having regular and irregular MC function, respectively. In addition to self‐report measures, alterations in urinary biomarkers (E1G, PdG, and LH) demonstrated that 92% of women had MC disruption during RT, while only 3% were classified as having nondisrupted cycles, and 5% being indeterminable. Taken together, these data are suggestive of significant MC dysregulation during RT. These findings align with prior investigations that documented MC disruption in 53% (Gifford et al., 2017) and 86% (Bozzini et al., 2023) in females during intensive military training. Furthermore, it is important to note that while 44% of female recruits reported having regular MCs during RT, female sex hormone metabolites showed that 92% of women experienced MC dysfunction during training. These results highlight discrepancies between self‐reported MC assessments and physiological biomarkers of reproductive function in women during rigorous military training. The physiological biomarkers assessed in this study provide objective data on HPOA function and can detect disruptions in the HPOA that bleeding patterns may miss. Similarly, Popp et al. (2024) observed disruptions in MC hormones (i.e., E1G, PdG, LH, etc.) in 75% of females undergoing U.S. Army Basic Combat Training (Popp et al., 2024). Moreover, in the 42% of trainees who reported having regular menses, only 12.5% had evidence of luteal activity (i.e., presumed ovulation), which is reflective of HPOA disruption in women who report having normal MCs (Popp et al., 2024).

In recruits who had MC disruption during RT, 87.5% experienced disruption onset during their first cycle, while 12.5% had disruption onset during their second cycle. These data suggest that MC disturbances occur more frequently during the early phases of USMC RT. Both the magnitude and quantity of physiological stressors (poor sleep quality, high training load, psychological distress) experienced by females undergoing arduous military training likely contributed to early onset of MC disruption. Furthermore, these findings suggest that HPOA function is highly sensitive to perturbations in physiological homeostasis and stress‐mediated neuroendocrine changes in women undergoing military training.

## 5 LIMITATIONS

There are limitations of this study that should be acknowledged. In general, conducting research in military settings renders it challenging to control external factors due to the high volume of recruits, unexpected injuries, and abrupt schedule changes. Saliva sampling time points were limited and selected based on phase changes and the recruits' activity schedule. Additionally, basal levels of stress biomarkers and urinary hormones were not assessed, as the first day of data collection took place 2 days prior to RT (i.e., pre‐RT). The use of self‐reported measures represents a limitation, as these data are susceptible to recall error and reporting bias. A total of 59 participants (49% of the sample) were excluded from the MC analyses due to incomplete 13‐week RT participation or missing urinary hormone data, representing a potential source of bias. Trends in urinary female sex hormone metabolites were assessed visually by research personnel, which introduces subjectivity and subtle or complex patterns may have been missed or misclassified. However, independent review by three trained researchers enhanced reliability via consensus, a pragmatic strength in this uncontrolled military environment characterized by high interindividual menstrual variability and operational stressors. This approach provides novel insights into hormone patterns in a military training environment that are unattainable in lab settings. We acknowledge that the lack of a control group may limit causal inference; however, access to a similar female military population not undergoing military training was not attainable. Nonetheless, the findings from our study are generalizable to female military populations undergoing rigorous training and provide new information on the physiological changes that can occur in operational environments.

## 6 CONCLUSION

In conclusion, this effort examined the effects of USMC RT on the physiological stress response (both SAM system and HPAA activity) and MC function in female Marine recruits. Specifically, high concentrations of sAA pre‐RT were observed, which may be indicative of anticipatory stress as recruits prepare for RT. Low levels of sAA exhibited during weeks 4, 7, and 10 compared to pre‐RT may be suggestive of SAM system recovery as recruits adapt to the compounding effects of stress characteristic of military training. High cortisol levels demonstrated during weeks 1 and 4 are reflective of heightened activation of the HPAA as recruits experience a sudden increase in allostatic load, whereas the lack of changes in cortisol documented at weeks 7, 10, and post‐RT may be suggestive of HPAA adaptation as recruits acclimate to operating in a multistressor environment. MC disruption documented in recruits via self‐report questionnaires and female sex hormone metabolites is reflective of HPOA suppression. Moreover, early onset of cycle disruption seen in a majority of recruits (87.5%) underscores the heightened sensitivity of the HPOA under conditions of high allostatic load experienced in female Marine recruits during rigorous military training. This study is one of few to investigate the physiological impacts of military training stress response (both SAM system and HPAA activity) and HPOA function in female recruits. Taken together, this study provides more knowledge on the physiological effects of military training in female military personnel, which is imperative for optimizing health and performance in this population.

## AUTHOR CONTRIBUTIONS

**Lauren M. Visconti:** Data curation; formal analysis; investigation; validation. **Andrea C. Givens:** Data curation; formal analysis; investigation; methodology; project administration; supervision; validation. **Laura J. Palombo:** Data curation; formal analysis; investigation; methodology; project administration; supervision; validation. **Brenda A. Niederberger:** Data curation; formal analysis; investigation; methodology; project administration; supervision; validation. **Emily B. Kloss:** Data curation; formal analysis; investigation; methodology; validation. **Daniel W. Bennett:** Data curation; formal analysis; investigation; methodology; validation. **Lorraine P. Turcotte:** Supervision; validation. **Karen R. Kelly:** Conceptualization; data curation; funding acquisition; investigation; methodology; project administration; resources; supervision; validation.

## FUNDING INFORMATION

Funding for this effort was provided by Defense Health Agency work unit N1627 under agreement number A2409‐021‐017‐067669. The recipient of this funding was Karen R. Kelly. The funding agency had no role in the design, collection, analysis, or interpretation of the data presented herein.

## CONFLICT OF INTEREST STATEMENT

There are no conflicts of interest to disclose. Leidos is a contractor supporting the Naval Health Research Center under government contract. This employment relationship does not create a conflict of interest for this publication. The findings reported in this research are presented independently of Leidos' corporate interests.

## DISCLAIMER

I am a military service member or employee of the U.S. Government. This work was prepared as part of my official duties. Title 17, U.S.C. §105 provides that copyright protection under this title is not available for any work of the US Government. Title 17, U.S.C. §101 defines US Government work as work prepared by a military service member or employee of the US Government as part of that person's official duties. This effort was supported by the Defense Health Agency under work unit no. N1627. The views expressed in this work are those of the authors and do not necessarily reflect the official policy or position of the Department of the Navy, Department of Defense, or the U.S. Government. The study protocol was approved by the Naval Health Research Center Institutional Review Board in compliance with all applicable Federal regulations governing the protection of human subjects. Research data were derived from an approved Naval Health Research Center Institutional Review Board protocol number NHRC.2020.0008.

## Data Availability

Source data for this study are not publicly available due to privacy or ethical restrictions. The source data are available to verified researchers upon request by contacting the corresponding author.
